# Cognitive Functioning of Geriatric Patients: Is Hypovitaminosis D the Next Marker of Cognitive Dysfunction and Dementia?

**DOI:** 10.3390/nu10081104

**Published:** 2018-08-16

**Authors:** Ewelina Łukaszyk, Katarzyna Bień-Barkowska, Barbara Bień

**Affiliations:** 1 Department of Geriatrics, Medical University of Bialystok, Fabryczna 27, 15-471 Bialystok, Poland; bien@umb.edu.pl; 2 Geriatric Ward, Hospital of the Ministry of Interior and Administration in Bialystok, Fabryczna 27, 15-471 Bialystok, Poland; 3 Institute of Econometrics, Warsaw School of Economics, Madalińskiego 6/8, 02-513 Warsaw, Poland; Katarzyna.Bien@sgh.waw.pl

**Keywords:** dementia, MMSE, vitamin D, vitamin B12, Timed Up and Go test (TUG)

## Abstract

The study objective is to investigate whether vitamin D is associated with the cognitive function of geriatric patients. This cross-sectional study involved 357 patients hospitalized in the geriatric ward who complained of memory problems (mean age: 82.3 years). The level of cognitive function was measured with the Mini-Mental State Examination (MMSE) and the clinical diagnosis of dementia was established according to the International Classification of Diseases (ICD-10) criteria. The serum 25-hydroxy vitamin D was measured with liquid chromatography-tandem mass spectrometry. The iterative Bayesian model averaging (BMA) procedure was applied to linear and logistic regression models in order to identify the best set of factors describing cognitive dysfunction and dementia, respectively. According to BMA, there is strong evidence that higher vitamin D levels, higher body mass index (BMI), and higher mobility function measured with the Timed Up and Go (TUG) test are independently associated with better cognitive performance and lower risk of dementia. Additionally, there is strong evidence that fewer years of education and lower vitamin B12 plasma levels independently describe worse cognitive performance. However, vitamin B12 levels higher than 800 pg/mL is negatively associated with the MMSE performance. Hypovitaminosis D in geriatric patients is an underrated marker of cognitive dysfunction and dementia.

## 1. Introduction

The global incidence of dementia is growing at an alarming rate. This condition is usually preceded by the mild cognitive impairment (MCI) that occurs in 10–20% of individuals older than 65 years of age [1]. Generally-accepted international guidelines recommend screening for vitamin B12 deficiency as well as hypothyroidism in patients with MCI or dementia [2]. In recent years, newer and more extensive studies describing the pleiotropic effects of vitamin D and the importance of its deficiency in many diseases, not strictly related to changes occurring in the skeletal system, have been published. These research issues concern cancer diseases [3,4,5], cardiovascular disease [6], diabetes [7], as well as mental illnesses [8,9,10]. The main sources of vitamin D are both oral intake and synthesis in the skin. 

There are many possible hypotheses as to why insufficient levels of vitamin D may lead to the development of cognitive dysfunction. The presence of vitamin D receptors has been demonstrated in Purkinje cells as well as the cerebral cortex and hippocampal neurons [11]. The biologically active metabolite of vitamin D enhances the expression of neurotrophins in the hippocampus, which is attributed to neuroprotective effects [12]. It has also been shown that vitamin D regulates oxidative stress mechanisms, calcium homeostasis, and immune system function in the central nervous system [13,14,15]. There is also evidence of vitamin D regulating the genetic expression of various neurotransmitters, including acetylcholine, dopamine, and serotonin [16]. Moreover, there is an observed link between hypovitaminosis D and brain atrophy [17,18]. Interestingly, patients with hypovitaminosis D are more likely to suffer strokes and diseases of the small vessels of the brain [19,20,21].

On the other hand, vitamin D may also have indirect effects on cognitive function and the cardiovascular system. It is well known that vitamin D regulates the renin-angiotensin-aldosteron system [22,23]. It also plays a protective role in cardiac remodeling [24], endothelial function [25], and arterial stiffness [26]. Recent studies have demonstrated that an independent relationship exists between arterial stiffness and cognitive impairment [27].

Moreover, midlife hypertension is a well-known modifiable risk factor for cognitive impairment in the elderly [28]. A recent study observed that supplementing vitamin D improved blood pressure control [29]. However, previous studies regarding the effects of vitamin D and the risk of dementia lead to contradictory results. This is likely due to differences in statistical analysis strategies as well as inclusion criteria and the adjustment of confounding factors. In one of the meta-analyses, the risk of cognitive impairment in the case of hypovitaminosis D was 2.4 times greater than those with adequate vitamin D status [30]. Conversely, in one of the latest articles, the authors denied the association of vitamin D and cognitive status in an 18-year follow-up [31].

The purpose of this study was to investigate, using the Bayesian Model Averaging approach, whether, after having controlled for other health-related factors, vitamin D describes the cognitive performance of geriatric patients. Moreover, we aimed to identify other potential medical predictors of functional decline in cognitive performance and dementia diagnosis. 

## 2. Materials and Methods 

This cross-sectional study involved 357 patients hospitalized in 2017 in the geriatric ward (21 beds) of a medium-sized hospital, drawing on a local population of more than 0.3 million. The anonymity of the patients was guaranteed and all patients were fully informed about the study and gave their consent. The retrospective study protocol was approved by the local Medical University Ethics Committee and conforms to the Helsinki Declaration.

A geriatric patient was defined as a person of advanced age with complex multimorbidity and who was referred to a geriatric ward by a primary care physician due to a recent deterioration of psychical and/or physical health [32]. All consecutive patients complaining about their memory worsening or exhibiting cognitive deficits in short-term memory screening tests were included in this analysis, irrespective of other co-existing ailments or conditions. All recruited patients underwent the Mini-Mental State Examination (MMSE) [33]. The MMSE is an effective and extensively used screening tool for cognitive impairment among older persons. It is also used to estimate the severity and progression of cognitive decline. This instrument relies on an 11-item questionnaire which measures several areas of cognitive function: Concentration or working memory; language and praxis; orientation; memory; and attention span. MMSE scores range from 0 to 30 with a higher score indicating better cognitive function. The clinical diagnosis of dementia was established based on multifaceted and in-depth examinations, including elements of the comprehensive geriatric assessment (CGA) [34], laboratory tests, neuropsychological investigation, and brain imaging, all conducted according to the International Classification of Diseases, 10th revision (ICD-10).

### 2.1. Response Variables

The MMSE score (ranging from 0 to 30), which represents a general and comprehensive measure of cognitive performance, was usually measured on the second day of hospitalization and was defined as a response variable in the statistical analysis. Moreover, the dichotomous variable indicating the clinical diagnosis of dementia (no vs. yes) according to the ICD-10 classification of the World Health Organization [35] was also defined as an alternative response variable in a second statistical model. Dementia was defined as a clinical syndrome involving a sustained and progressive loss of memory and/or other cognitive functions of significant severity, causing dysfunctions in daily living in patients with no disturbance of consciousness. 

The dual outlook (two models) used in our study results from ambiguous and inconclusive scoring on the MMSE that should be used as a screening tool only. The dementia diagnosis of any origin (Alzheimer’s disease, vascular dementia, Lewy bodies dementia, Parkinson’s type, or frontotemporal dementia) was confirmed by the criteria for this disease, after ruling out possible abnormalities mimicking the condition, like depressive pseudodementia, delirium or confusion, mild cognitive impairment, or reversible causes of dementia. 

### 2.2. Patient Characteristics as Potential Explanatory Variables

Many different characteristics of geriatric patients were investigated as potential explanatory variables (potential predictors) for both cognitive function and the risk of dementia diagnosis (Table 1). These encompassed the sociodemographic data: Age; gender; education; place of living (urban vs. rural); living alone vs. with family member or others; and anthropometric data such as body mass index (BMI) in kg/m^2^. The measure of a patient’s blood pressure (BP) in a supine position was taken in the morning one day after admission, irrespective of prescribed medication.

After an overnight fasting, blood samples were collected in all subjects during a morning enrolment session. Hematological measurements were made using fresh venous blood with ethylene diamine tetraacetic acid (EDTA) and clotted blood. Serum 25-hydroxy vitamin D, (25(OH)D) in ng/dL (1 nmol/L = 0.4 ng/mL) were made by liquid chromatography-tandem mass spectrometry (automated system). The categories of serum vitamin D levels were additionally defined as follows: Severe deficiency (<20 ng/mL); moderate deficiency (20–29.9 ng/mL); slight deficiency (30–49.9 ng/mL, and normal range (50 or more ng/mL). Other laboratory tests included: hemoglobin (in g/dL); lymphocytes (count per mm^3^); plasma sodium and potassium (both in mmol/L); thyrotropin stimulating hormone (TSH) in µIU/L; C-reactive protein (CRP) in g/dL; plasma albumin (in g/dL); fasting glucose (mg/mL); glomerular filtration rate (GFR) according to the Cockcroft Gault formula per 1.73 m^2^; plasma vitamin D (ng/mL); and plasma vitamin B12 (pg/mL). All were measured using standard laboratory methods in a certified local central laboratory.

The CGA was routinely carried out in all patients by the geriatric team (geriatricians, nurses, physiotherapist, and psychologist) on the first or second day after admission. To this end, the Timed Up and Go (TUG) test was performed to determine a patient’s mobility [36]. The TUG test measures the time (in seconds) taken by a patient to rise from a chair and walk three meters, turn around, walk back to the chair, and sit down (the use of an assistance device was allowed if needed). The speed of the TUG performance was recalculated for all patients; for bedridden patients, the value 0 m/s was ascribed.

Multimorbidity was based on the sum of the 18 most prevalent discharge clinical diagnoses, defined as follows: Depressive disorders; delirium or confusion during hospital stay; hypertension (treated or newly recognized); osteoarthritis (radiological and clinical presentation); presence of anemia (hemoglobin < 12 g/dL in females or < 13 g/dL in males); diabetes mellitus (treated or newly diagnosed); atrial fibrillation (paroxysmal, persistent, or permanent); congestive heart failure; ulcer disease; chronic kidney disease (glomerular filtration rate according to the Cockcroft-Gault formula < 50 mL/min/1.73 m^2^); Parkinson’s disease; cerebrovascular disease (confirmed with the CT brain scans in transversal plain or carotid artery Doppler test performed and described by certified hospital laboratories); liver disorders; thyroid dysfunction; benign prostatic hyperplasia; neoplastic disease; chronic obstructive pulmonary disease; and/or connective tissue disease.

The broad range of characteristics were included into the model due to their potential contribution to cognitive problems, e.g., inflammatory, metabolic, hemodynamic, neoplastic, hormonal, or of other origin.

### 2.3. Statistical Analysis

First, univariate analyses were performed to test the significance of the relationship between each of the two response variables measuring the patient’s cognitive status and their potential sociodemographic or health-related predictors. Linear regression models were applied for the MMSE score and the logistic regression models for the dichotomous indicator of dementia diagnosis. The explanatory variables were log-transformed if such a nonlinear functional form resulted in a better goodness-of-fit. A binary indicator of vitamin B12 > 800 pg/mL was introduced as an additional explanatory variable because elevated levels of vitamin B12 can indicate serious underlying pathologies, including life-threatening conditions [37,38]. Standard errors of the parameter estimates and the corresponding *p*-values were derived using the robust Huber formula [39].

Second, computation-intensive variable selection methods were used to identify the best multiple variable models for the MMSE score and the dementia diagnosis. To make our results statistically robust, the stepwise backward variable selection method (with the significance level set as *p*-value < 0.05) was also accompanied by the Bayesian model averaging (BMA) procedure [40]. Statistical inference based on a single model derived with the stepwise selection algorithm can be risky because the ambiguity about performed model selection does not dilute information about obtained coefficients and predictions [41,42]. Contrary to the classical stepwise selection procedures, BMA constitutes a very coherent mechanism that corrects for the uncertainty of the underlying model [43,44]. This allows us to assess whether any redundant explanatory variable has been inappropriately selected as a significant predictor of the MMSE score or dementia. The BMA approach relies on appropriate weighted averaging over all possible models that differ with respect to subsets of included explanatory variables (combinations of potential predictors). Interpretable results from the BMA procedure comprise of (1) the posterior mean parameter, *E*(*β*|*D*), measuring the direction and strength of the relationship between the variables under study and (2) the posterior probability that this parameter is nonzero, *P*(*β*≠0|*D*), where *D* denotes the data. The interpretation of obtained values can be performed as follows [42]. If *P*(*β*≠0|*D*) < 0.5, there is evidence against an independent association between a given explanatory variable and the MMSE or the dementia diagnosis; if 0.5 < *P*(*β*≠0|*D*) < 0.75, there is weak evidence of an association; if 0.75 < *P*(*β*≠0|*D*) < 0.95, there is positive evidence; if 0.95 < *P*(*β*≠0|*D*) < 0.99, there is strong evidence and if 0.99 < *P*(*β*≠0|*D*) < 1, there is very strong evidence that a variable is independently linked with the MMSE or the diagnosis of dementia. For a final variable selection, the iterative BMA was performed [45] where only explanatory variables with posterior probability greater than 0.3 were retained in the model averaging procedure. Statistical analyses were performed with STATA software version 15.0 (StataCorp LLC, College Station, TX, USA) and R software (version 3.4.2, R Foundation for Statistical Computing, Vienna, Austria) with BMA package for the Bayesian model-averaging [46].

## 3. Results

The sample of patients included 236 women and 121 men (mean age 82.3, ranging from 62–102 years of age). The basic descriptive statistics of the data are presented in Table 1. The average length of a hospital stay was 6.8 days, from 2 to 42 days. All patients enrolled to the study presented worsening of memory and other complaints: Diffuse or focal pain of musculoskeletal system–56.5%; dizziness–44.8%; weakness–42.5%; fall(s) in previous year–54.1%; recent syncope–11.3%. Comorbidity out of the 18 defined and most prevalent discharged diagnoses accounted for nearly five conditions per patient, including hypertension–69.2%; cerebrovascular disease–60.5%; depression–57.1%; degenerative joint disease–56.0%, dementia of any origin–42.5%; anemia–36.9%; diabetes mellitus–35.3%; congestive heart failure–26.1%, atrial fibrillation–25.2%, delirium–23.2%; ulcer disease–22,1%; Parkinson’s disease or extrapyramidal disorders–13.4%, cancer–11.5%, and other. All of the co-existing conditions, along with its complications and medications used, prove a great heterogeneity of geriatric patients that may influence a clinical presentation of any stage of cognitive dysfunction.

**Figure 1:**
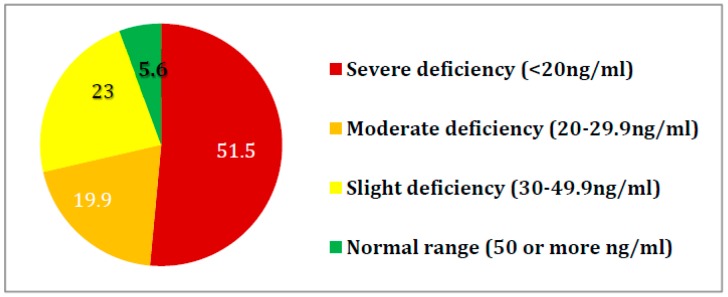
Structure of vitamin D level in geriatric patients (*n* = 357, in %).

Median of vitamin D concentration in the studied population remained below the normal level. Structure of vitamin D level in geriatric patients is presented in Figure 1. Patients with dementia diagnosis in 26% of cases supplemented vitamin D prior to the study, and those without dementia in 37%.

According to the univariate models, a MMSE score is negatively related to the patient’s age, urban place of living, sodium level, and the number of coexisting medical conditions (Table 2). On the other hand, the patient’s level of education, potassium and albumin levels, GFR, BMI, lymphocyte count, levels of glucose, vitamin D and B12, as well as the speed of performing the TUG test, proved to be positively correlated with an MMSE score. The last six variables are also negatively correlated with the risk of dementia. The relationship between cognitive function and the plasma vitamin B12 is nonlinear. If vitamin B12 levels exceed 800 pg/mL, there is a decline in the MMSE score and an increase in the risk of dementia. The likelihood of a dementia diagnosis also increases in line with age and the number of medical conditions present in geriatric patients.

In the multiple variable models obtained using stepwise backward selection algorithm, the jointly significant variables describing a higher MMSE score and lower dementia risk are as follows: BMI, natural logarithms of vitamin D and B12 levels, and motor function measured by the natural logarithm of the TUG test speed. The functional decline in cognitive performance is also shown to be alleviated by the patient’s years of education, whereas the risk of dementia is shown to be negatively related to the lymphocyte count and positively associated with the increase in hemoglobin level as a possible marker of dehydration.

The Bayesian averaging approach confirms very strong evidence (*P*(*β*≠0) = 1|*D*) that after having controlled for other factors the mobility function assessed using the log speed of performing the TUG test is positively associated with better cognitive performance, measured with MMSE score, (*E*(*β*|*D*) = 14.950) and negatively associated with risk of dementia (*E*(*β*|*D*) = −3.411) (Table 3).

**Figure 2:**
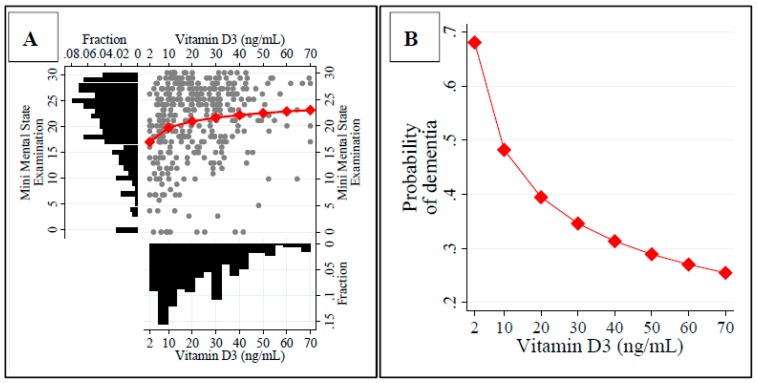
Relationship between the vitamin D level and the score of the Mini Mental Score Examination (**A**) or the diagnosis of dementia (**B**) in an average geriatric patient: Results of the Bayesian model averaging approach.

There is also very strong evidence (*P*(*β*≠0|*D*) = 1) that a higher education level (*E*(*β*|*D*) = 0.422), BMI (*E*(β|*D*) = 0.306), as well as the levels of both vitamin B12 up to 800 pg/mL (*E*(*β*|*D*) = 3.315) and D (*E*(*β*|*D*) = 1.694) are independently positively linked to MMSE score. Higher BMI is also shown to be an additional factor that might alleviate the risk of dementia (*E*(*β*|*D*) = −0.072; *P*(*β*≠0|*D*) = 1). However, contrary to the results obtained with the standard backward selection method, there is weak evidence that dehydration is independently associated with the exacerbated cognitive impairment, lowering the MMSE score (*P*(*β*≠0|*D*) = 0.568 for the sodium level) and increasing the risk of dementia (*P*(*β*≠0|*D*) = 0.730 for the hemoglobin level). The relationship between vitamin D and the MMSE score is nonlinear and the largest in value for patients with severe hypovitaminosis. If the vitamin D level increases from 2 up to 20 ng/mL, the average MMSE score increases from 17 to 21 points (Figure 2, Panel A). Having controlled for other covariates, there is strong evidence that vitamin D is associated with the diminished risk of dementia (*P*(*β*≠0|*D*) = −0.514; *P*(*β*≠0|*D*) = 0.984)) (Figure 2, Panel B).

## 4. Discussion

As the population ages, a major clinical issue and key public health problem is the progression of cognitive dysfunction and dementia [47]. Therefore, there is an urgent need to recognize the exhaustive set of potentially reversible factors that might lead to dementia in order to intervene in a timely fashion and slow down the process of cognitive decline. This study aims to identify the best set of indicators, both for functional decline in cognitive performance and the late-onset of dementia, the latter corresponding mostly to Alzheimer’s disease with a component of cerebrovascular disorders.

Our study confirms the well-known findings that the functional decline in cognitive performance is exacerbated for low-educated adults and the risk of dementia increases with age. The novelty of our study lies in evidence of nonlinear dependence between the beneficial levels of vitamin B12 and MMSE scores. According to BMA, there is strong evidence that hypervitaminosis B12 (cut off point > 800 pg/mL) is independently associated with the deterioration of the MMSE outcome. The high serum vitamin B12 is still an under recognized anomaly that predominately encompasses severe and often critical diseases, such as neoplasms, hematological malignancies, liver, or kidney diseases; however, the internal mechanisms between these conditions are not fully understood [38]. Recently, Cappello S. et al. have revealed an independent association between elevated vitamin B12 concentrations (>1000 pg/mL) and in-hospital mortality in adult patients at nutritional risk [48]. Therefore, these novel and interesting findings need further elucidation.

According to our study, there is very strong evidence that higher vitamin D levels, a higher BMI, or a better TUG performance independently describes better cognitive status and lower risk of dementia in geriatric patients. Overall, these factors can be treated as markers of good physical and nutritional status. The latter is considered a key factor in mitigating vascular dementia [49]. As late-onset Alzheimer’s disease often occurs with small cerebral vessel disease, the appropriate supply of macro- and micronutrients is of high priority in older adults. The great importance of vitamin D in atherosclerosis is already proven, as this vitamin exerts a variety of favorable effects on endothelial dysfunction [50]. Measured with MMSE, our study also shows that the lower the BMI in geriatric and comorbid patients, the higher risk of dementia and the more severe the functional decline in cognitive performance. The underlying mechanism linking BMI and cognition is not fully understood and requires further research, however some studies have shown that higher BMI in elderly patients is associated with a lower risk of cognitive impairment and dementia [51], as well as longer survival [32]. In the latest prospective study, low baseline BMI might be a useful marker for identifying individuals at increased risk for Alzheimer’s Disease in the Mild Cognitive Impairment subjects [52].

The recent results of meta-analysis on dietary patterns in older adults show a beneficial impact of vitamin D levels mitigating the risk of dementia and cognitive impairment [53], and are consistent with other studies regarding the relationship between vitamin D levels and dementia [53,54,55,56]. The first case-controlled study describing the correlation between Alzheimer disease and vitamin D was carried out in 1989 [54]. In 2006, Wilkins et al. [55] published the first cross-sectional study of 80 ambulatory elderly patients and showed that hypovitaminosis D was correlated with poorer performance on the Short Blessed Test. Since then, numerous cross-sectional studies have been put forward. However, to the best of our knowledge, our study is the first conducted with very advanced-age multimorbid patients hospitalized in a geriatric ward. Moreover, no previous study investigating the links between dementia and vitamin D has considered so many diverse, potentially confounding sociodemographic and health-related explanatory factors. The additional merit of our analysis lies in constructing two different statistical models for distinct measures of cognitive decline: (1) Dysfunction of cognitive performance evaluated by the MMSE score, which can represent transient cognitive deterioration during depression or other diseases; as well as (2) a clinical dementia diagnosis of different origin. This latter case comprises different types of dementia as Alzheimer disease, vascular dementia, and other mixed disorders. Although the cross-sectional design of our study precludes defining strictly causal relationships, our results are in line with the recent findings in longitudinal studies that suggest the association between hypovitaminosis D and cognitive impairment [56,57]. However, the number of intervention studies that reported a positive effect of vitamin D supplementation on cognitive performance is low [58,59].

Recently, it has been shown that serum vitamin D was lower in MCI and various stages of Alzheimer’s disease. Accordingly, vitamin D status could be treated as a useful biomarker for predicting and diagnosing MCI and Alzheimer disease [60]. This is in line with the results of a former study that indicated a positive association between MMSE test results and vitamin D concentration [61], although no correlation between MMSE and vitamin B12 levels has been observed. In contrast, we have demonstrated that both very low and considerably elevated serum vitamin B12 concentration is negatively associated with the MMSE score, a finding that remains in accordance with generally accepted guidelines to include the vitamin B12 status in the standard evaluation of cognitive decline. On the other hand, we have also documented the weak evidence that hypovitaminosis B12 might increase the risk of the clinical diagnosis of dementia, whilst low vitamin D concentration—especially below 20 ng/mL—is shown to be an undisputable additional factor that is independently linked to the risk of both dementia and functional decline in cognitive performance.

Like vitamin D status, higher physical activity promotes cognitive functioning in older adults [62]. A very recent study by Lee et al. [63] has revealed that impaired TUG performance was associated with increased risk of all causes of dementia, which remains in line with our results. According to BMA, the speed of performing the TUG test is the only variable with very strong evidence of (1) an independent negative association with the risk of dementia and (2) an independent positive association with the MMSE score. This link between TUG and cognitive performance has not been studied so far. Although the pathophysiological impact of gait disorders and cognitive dysfunction is not well understood [64], our findings suggest that TUG may be an easily accessible test used to detect patients at risk of dementia.

The limitation of our study lies in its cross-sectional design that impedes conclusions about direct causality relations between the variables under study. Moreover, the relatively small sample size, in the absence of unified diagnostic criteria for vascular dementia in geriatric patients, precluded exploring the relationship between vitamin D and types of dementia. To overcome this relatively small sample size and to reduce the potential incidence of coincidental relationships, the BMA was applied, which, due to the variable selection procedure, corrects for the model’s uncertainty. Additionally, it is shown to outperform stepwise approaches at predicting an event of interest [42]. The application of the BMA method fits into recent discussions about the necessity of improving statistical inference on a large scale [65] and is considered as a possible remedy for observed misusage, overreliance, and misinterpretation of empirical results obtained with statistical significance thresholds defined as *p* < 0.05 [66].

## 5. Conclusions

In conclusion, the results presented in this study prove that, in addition to lower mobility and undernutrition, hypovitaminosis D in geriatric patients constitutes an underrated marker for worse cognitive performance and dementia. Continuous monitoring of vitamin D status should be recommended in everyday clinical practice in all older persons, and any deficits should be supplemented. 

## Figures and Tables

**Table 1:** Sociodemographic and health-related characteristics of geriatric inpatients (*n* = 357).

Characteristics	Dichotomous Variables		Continuous or Polytomous Variables				
	*n*	%	Min	Max	Median	Mean	SD
Dependent variables							
MMSE (0–30)			0	30	22	20.86	7.06
Dementia diagnosis (yes)	152	42.5					
Independent variables							
Age (years)			62	102	83	82.30	6.67
Male (yes)	121	33.9					
Number of years in education			0	23	9	9.31	4.29
Urban place of living (yes)	266	74.5					
Living alone (yes)	115	32.2					
Fall(s) in last year (yes)	193	54.1					
BMI (kg/m^2^)			15.9	67.89	27.47	28.29	5.94
sBP (mmHg)			87	201	130	130.96	19.54
dBP (mmHg)			41	102	66	67.33	11.19
Hemoglobin (g/dL)			7.08	17.7	12.79	12.58	1.69
Lymphocytes (count per mm^3^)			570	9400	1650	1763.17	801.39
Sodium (mmol/L)			116.0	149.0	140.0	139.94	3.19
Potassium (mmol/L)			3.04	6.66	4.34	4.40	0.50
TSH (µIU/L)			0.003	71.65	1.19	1.81	4.86
CRP (g/L)			0.0	374	2	9.500	29.14
Albumin (g/dL)			2.38	4.9	4.1	3.9	0.4
Fasting glucose (mg/mL)			36.0	406.0	99.0	107.28	30.81
GFR (in mL/min/1.73 m^2^)			6.35	169.54	56.72	61.38	25.53
Total cholesterol (mmol/L)			75.0	315	170	174.74	46.35
Vitamin D (ng/mL)			2.0	70.0	19.3	22.31	14.99
Vitamin B12 (pg/mL)			50.0	2000	335	390.58	248.97
Speed of TUG (m/s)			0	2.07	0.32	0.35	0.26
Sum of conditions out of top 18			0	11	5	5.29	1.92

SD: standard deviation; MMSE: Mini-Mental State Examination; BMI: body mass index; sBP: systolic blood pressure; dBP diastolic blood pressure; TSH: thyrotropin stimulating hormone; CRP: C-reactive protein blood test; GFR: glomerular filtration rate; I-ADL: Instrumental activity of daily living; TUG: Timed Up and Go Test.

**Table 2:** The association between the sociodemographic or health-related characteristics of geriatric inpatients and the MMSE score/the clinical diagnosis of dementia. The results are from univariate and multiple regression models.

Explanatory Variable	Univariate Models				Multiple Variable Models			
	MMSE		Dementia Diagnosis		MMSE		Dementia Diagnosis	
	Coefficient (95% CI)	*p*-Value	OR (95% CI)	*p*-Value	Coefficient (95% CI)	*p*-Value	OR (95% CI)	*p*-Value
Age (years)	−0.327 (−0.428; 0.226)	<0.001	1.079 (1.042; 1.119)	<0.001	-	-	1.043 (1.004; 1.083)	0.031
Male (yes)	1.266 (−244; 2.776)	0.100	0.973 (0.625; 1.518)	0.907	-	-	-	-
Number of years in education	0.556 (0.401; 0.711)	<0.001	0.959 (0.912; 1.009)	0.109	0.426 (0.300; 0.553)	<0.001	-	-
Urban place of living (yes)	−1.785 (−3.547;0.022)	0.047	0.899 (0.554; 1.460)	0.669	-	-	-	-
Living alone (yes)	1.327 (−0.097; 2.750)	0.068	0.554 (0.348; 0.881)	0.013	-	-	-	-
Fall(s) in last year (yes)	−0.095 (−1.589; 1.398)	0.900	1.088 (0.713; 1.658)	0.695	-	-	-	-
BMI (kg/m^2^)	0.228 (0.120; 0.334)	<0.001	0.953 (0.918; 0.988)	0.010	0.311 (0.211; 0.411)	<0.001	0.933 (0.888; 0.979)	0.005
sBP (mmHg)	0.031 (−0.094; 0.047)	0.089	0.993 (0.998; 1.036)	0.228	-	-	-	-
dBP (mmHg)	−0.024 (−0.005; 0.066)	0.514	1.017 (0.983; 1.004)	0.074	-	-	-	-
Hemoglobin (g/dL)	0.163 (−0.308; 0.634)	0.497	1.087 (0.959; 1.233)	0.191	-	-	1.256 (1.083; 1.457)	0.003
Ln (Lymphocytes) (count per mm^3^)	2.582 (0.703; 4.461)	0.007	0.359 (0.199; 0.646)	0.001	-	-	0.473 (0.240; 0.933)	0.031
Sodium (mmol/L)	−0.232 (−0.464; −0.000)	0.050	1.063 (0.992; 1.138)	0.084	−0.231 (−0.425; −0.038)	0.019	-	-
Potassium (mmol/L)	1.579 (0.103; 3.055)	0.036	0.714 (0.463; 1.100)	0.127	-	-	-	-
Ln (TSH)	−0.258 (−1.088; 0.571)	0.540	1.058 (0.833; 1.345)	0.641	-	-	-	-
CRP (g/L)	−0.013 (−0.040; 0.014)	0.343	0.994 (0.986; 1.002)	0.184	-	-	-	-
Albumin (g/dL)	0.079 (0.011; 0.147)	0.022	0.634 (0.376; 1.071)	0.089	-	-	-	-
Ln (Fasting glucose) (mg/mL)	4.127 (1.221; 7.033)	0.005	0.273 (0.102; 0.728)	0.010	-	-	-	-
GFR (mL/min/1.73 m^2^)	0.051 (0.026; 0.075)	<0.001	0.992 (0.984; 1.000)	0.062	-	-	-	-
Total cholesterol (mmol/L)	1.008 (−2.002; 4.017)	0.510	1.124 (0.510; 2.475)	0.772	-	-	-	-
Ln (Vitamin D) (ng/mL)	2.327 (1.412; 3.242)	<0.001	0.586 (0.446; 0.771)	<0.001	1.713 (0.922; 2.502)	<0.001	0.600 (0.444; 0.809)	0.001
Ln T(Vitamin B12) (pg/mL)	3.584 (1.654; 5.513)	<0.001	0.486 (0.290; 0.816)	0.006	3.219 (1.597; 4.841)	<0.001	0.438 (0.245; 0.784)	0.006
Vitamin B12 > 800 pg/mL	−5.962 (−9.882; −2.043)	0.003	4.794 (1.512; 15.204)	0.008	−5.159 (−8.439; −1.879)	0.002	5.764 (1.576; 21.083)	0.008
Ln (TUG speed+1) (m/s)	17.589 (13.684; 21.495)	<0.001	0.029 (0.007; 0.124)	<0.001	14.905 (11.375; 18.435)	<0.001	0.036 (0.007; 0.193)	<0.001
Sum of conditions out of top 18	−0.472 (−0.840; −0.102)	0.012	1.121 (1.004; 1.252)	0.043	-	-	-	-

CI, confidence interval; OR, odds ratio; BMI, body mass index; sBP: Systolic blood pressure; dBP: diastolic blood pressure; CRP: C-reactive protein blood test; GFR: Glomerular filtration rate; I-ADL: Instrumental activity of daily living; TUG: Timed Up & Go Test.

**Table 3:** The explanatory variables for the MMSE score and the diagnosis of dementia. The results from the iterative Bayesian model averaging procedure.

	MMSE		Dementia Diagnosis	
Predictors	*E*(*β*|*D*) (*SD*(*β*|*D*)	*P*(*β*≠0|*D*) *	*E*(*β*|*D*) (*SD*(*β*|*D*)	*P*(*β*≠0|*D*)
Age (years)	-	-	0.024 (0.028)	0.501
Number of years in education	0.422 (0.070)	1	-	-
BMI (kg/m^2^)	0.306 (0.050)	1	−0.072 (0.028)	0.955
Hemoglobin (g/dL)	-	-	0.147 (0.110)	0.730
Sodium (mmol/L)	−0.133 (0.136)	0.568	-	-
Ln (Vitamin D) (ng/mL)	1.694 (0.380)	1	−0.514 (0.171)	0.984
Ln (Vitamin B12) (pg/mL)	3.315 (0.754)	1	−0.380 (0.453)	0.493
Vitamin B12 > 800 pg/mL	−5.150 (1.860)	0.961	0.700 (0.948)	0.418
Ln (Speed of TUG) (m/s)	14.950 (1.687)	1	−3.411 (0.831)	1

* The posterior probability that a coefficient is nonzero given the data.
